# Adipose Tissue-Derived Mesenchymal Stem Cell-Derived Exosomes Promote Wound Healing and Tissue Regeneration

**DOI:** 10.3390/ijms241310434

**Published:** 2023-06-21

**Authors:** Jun Ho Lee, Yu Jin Won, Hail Kim, Minji Choi, Esther Lee, Bumsik Ryoou, Seok-Geun Lee, Byong Seung Cho

**Affiliations:** 1 ExoCoBio Exosome Institute (EEI), ExoCoBio Inc., STE 306, 19 Gasan digital 1-ro, Geumcheon-gu, Seoul 08594, Republic of Korea; junho.lee@exocobio.com (J.H.L.); yujin.won@exocobio.com (Y.J.W.); esther.lee@exocobio.com (E.L.); bumsik.ryoou@exocobio.com (B.R.); 2 Graduate School, Kyung Hee University, Seoul 02447, Republic of Korea; khi3598@khu.ac.kr (H.K.); minjichoi8466@khu.ac.kr (M.C.); 3 BioNanocomposite Research Center, Kyung Hee University, Seoul 02447, Republic of Korea; 4 Clinical Research Institute, Kyung Hee University Medical Center, Seoul 02447, Republic of Korea

**Keywords:** adipose tissue-derived mesenchymal stem cells, exosomes, hyaluronic acid, wound healing, tissue regeneration

## Abstract

Wound healing is a complex process involving cell proliferation, migration, and extracellular matrix (ECM) remodeling. Extracellular vesicles (EVs) or exosomes derived from adipose tissue-derived stem cells (ASCs) are emerging as promising alternatives to cell therapy for advanced wound healing. Hyaluronic acid (HA), a major component of the skin ECM, is widely utilized in wound dressings and dermal fillers. This study aimed to investigate the effects of ASC-derived exosomes (ASC-EXOs) on human dermal fibroblasts (HDFs) and their potential combination with HA in in vivo wound healing and dermal filler models. In HDFs, ASC-EXOs increased cell proliferation and migration. ASC-EXOs also upregulated the expression of genes involved in cell proliferation and wound healing while stimulating collagen production in HDFs. In a porcine wound healing model, topical treatment with a combination of HA and ASC-EXOs led to higher wound closure rates compared to HA alone. Histological examination showed increased re-epithelialization and collagen type III deposition in wounds treated with the combination of HA and ASC-EXOs. In a mouse dermal filler model, tissues injected with the combination of HA and ASC-EXOs exhibited thicker tissue layers, increased vascularization, enhanced infiltration of myofibroblasts, and higher levels of collagen III and collagen fiber content compared to HA alone. These findings suggest that ASC-EXOs have beneficial effects on cell proliferation, migration, and gene expression related to wound healing, and they may accelerate wound closure and promote tissue regeneration. Furthermore, the combination of HA and ASC-EXOs may enhance wound healing and tissue remodeling, indicating its potential for both clinical and regenerative aesthetic applications in skin repair and regeneration.

## 1. Introduction

Exosomes are nanosized (100 nm to 200 nm) extracellular vesicles (EVs) that are naturally secreted by most cells to facilitate cell–cell communication by transporting both genetic and molecular materials to neighboring cells and tissues [1,2]. The features and characteristics of exosomes have been extensively described in the previous literature; to oversimplify, these nanovesicles are well known to be enriched in proteins, lipids, and nucleic acids, serving as critical mediators of key biological processes in both cells and tissues through intracellular signaling mechanisms [3]. While the full extent of their characteristics and function remains to be elucidated, the collaborative efforts of the International Society of Extracellular Vesicles (ISEV) have outlined the minimal requirements for the definition, isolation, characterization, production, and storage condition for their application in many nonclinical and clinical settings that include cell-free therapy and advanced wound care [4,5].

Exosomes are purified from the secretomic milieu of cell cultivations through various purification methods that often include size exclusion chromatography, ultracentrifugation, density gradient filtration, tangential flow filtration, and affinity chromatography [6]. In tandem, previous studies have shown that the isolated vesicles possess therapeutic properties relevant to the derived cell types such as dendritic cells, mesenchymal stem cells (MSCs), adipose-derived stem cells, and pluripotent stem cells [7].

MSC-derived exosomes were reported to contribute to all stages of wound healing through the induction of rapid re-epithelialization, promotion of collagen maturity, and decreased scar formation in acute skin wounds due to the proangiogenic and anti-inflammatory properties inherited from parental cells [8,9]. Similarly, adipose tissue-derived mesenchymal stem/stromal cell-derived exosomes (ASC-EXOs) are also known to be involved in tissue regeneration and wound healing by modulating inflammation, angiogenesis, and extracellular matrix (ECM) reconstruction [10,11,12]. Most importantly, both MSC- and ASC-derived exosomes appear to inherit specific functions that resemble the paracrine effects of the parental stem cells such as immunomodulation [13,14] without the associated risks of cell transplantation which is highly advantageous for clinical use [10]. Previously, we showed that ASC-EXOs promoted epidermal barrier repair in atopic dermatitis by inducing the de novo synthesis of ceramides. Furthermore, we reported a safe toxicological profile in relation to skin sensitization, photosensitization, eye and skin irritation, and acute oral toxicity when administrated as a topical treatment [15,16].

On the basis of the promising results of mesenchymal stem cell exosomes in skin wound healing from previous studies, we aimed to further enhance their effects by combining them with a commonly used biomaterial for wound healing and regeneration. Hyaluronic acid (HA), a non-sulfated glycosaminoglycan and a major component of the skin ECM, is widely recognized for its valuable properties in wound healing and regeneration. These properties include high cellular compatibility, moisture retention, antiaging, favorable cell interaction, and CD44 targeting, making it a frequently utilized biomaterial for wound dressings and dermal fillers [17,18,19]. Therefore, in this study, we investigated the effects of combining ASC-EXOs with HA. We examined the effects of ASC-EXOs on human dermal fibroblasts (HDFs) in vitro, as well as their combination with HA in a mouse dermal filler model. Additionally, we investigated the combination effects in a porcine wound healing model. It is worth noting that most in vivo studies involving exosomes have been conducted in rodent models with acute cutaneous wounds, which exhibit notable anatomical, physiological, and tissue regeneration differences from human skin [20,21,22]. Consequently, we chose the porcine model to provide a more clinically relevant evaluation of the effects on wound healing.

## 2. Results

### 2.1. Effects of ASC-EXOs in Human Dermal Fibroblasts (HDFs)

**Figure 1:**
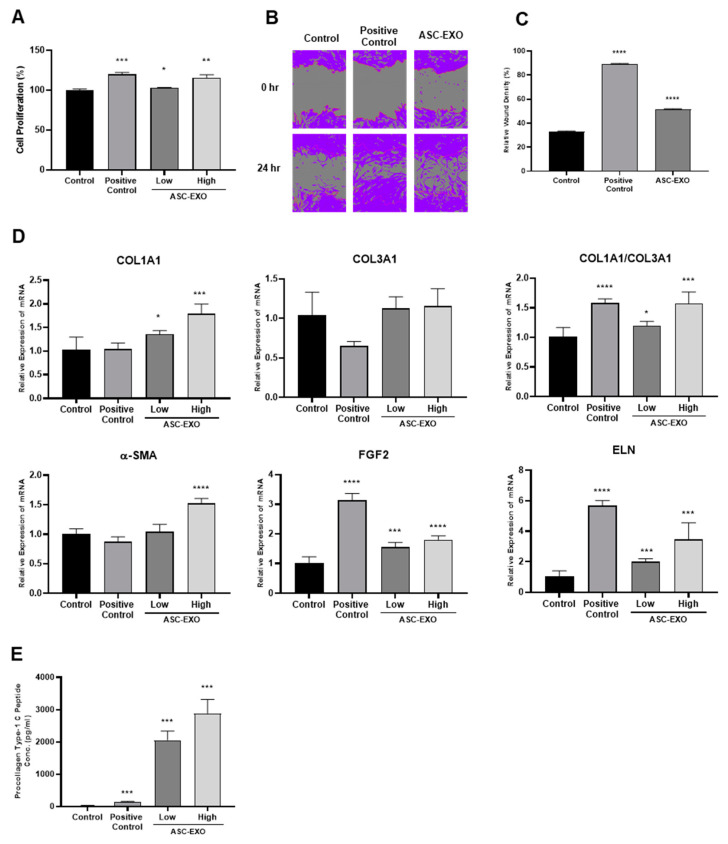
Effects of ASC-EXOs on HDF proliferation, migration, and collagen synthesis. (**A**) HDFs were treated with positive control or ASC-EXOs in low (3 × 10^9^ particles/mL) and high (1.5 × 10^10^ particles/mL) concentrations for 24 h. Cell proliferation rate was determined by CCK-8 assays. (**B**) HDFs with 100% confluency in wells of a 96-well plate were scratched, and the migration results were photographed at 0 and 24 h. Purple indicates the cell area. (**C**) The scratch-wound assay recovery rate is presented as the percentage closure. (**D**) HDFs were treated with ASC-EXOs for 24 h, and mRNA expression levels of genes encoding collagen type I and type III, α-SMA, FGF2, and elastin (ELN) were analyzed by normalization to the *GADPH* levels. (**E**) Procollagen type 1 C-peptide concentration was determined after 24 h treatment of ASC-EXOs in HDFs. The data are shown as the mean ± standard deviation (SD) (*n* = 3) with significance at * *p* < 0.05, ** *p* < 0.01, *** *p* < 0.001, and **** *p* < 0.0001.

In vitro evaluation of ASC-EXOs was conducted in HDFs to assess their potential effects on cell proliferation, migration, and ECM remodeling. ASC-EXOs increased HDF cell proliferation in a dose-dependent manner, with the ASC-EXO group showing an average of a 120% increase in total proliferation compared to the control (Figure 1A), indicating its stimulatory effect on cell proliferation. The scratch-wound assay revealed that ASC-EXOs promoted the migration of HDFs into the cell-free area compared to the negative control (Figure 1B,C). These results suggest that ASC-EXOs accelerate wound closure, which can occur, in part by promoting skin cell proliferation during wound healing. ASC-EXOs also induced the upregulation of mRNA expression levels of genes encoding collagen, α-smooth muscle actin (α-SMA), fibroblast growth factor 2 (FGF2), and elastin, which are known to be involved in cell proliferation and wound healing in the skin (Figure 1D). Furthermore, collagen production in HDFs increased significantly in a concentration-dependent manner under ASC-EXO treatment (Figure 1E). Collectively, these results suggest that ASC-EXOs have beneficial effects on cell proliferation, migration, and gene expression related to wound healing in HDFs, and they may have the potential to accelerate wound closure and promote tissue regeneration in the context of wound healing and skin repair.

### 2.2. Effects of a Combination of HA and ASC-EXOs in a Porcine Wound Healing Model

**Figure 2:**
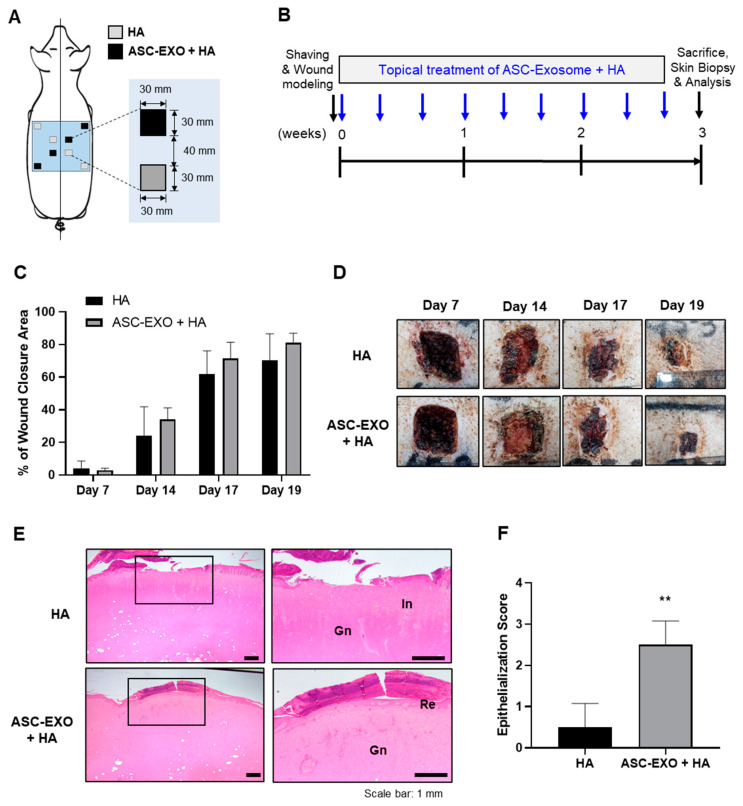
Effects of ASC-EXOs on wound closure and re-epithelialization in a porcine acute wound healing model. (**A**) Schematic diagram depicting the skin wounds and the topical treatments in a specific pathogen-free (SPF) mini pig. (**B**) Schedule of wound modeling, topical treatment, and tissue collection. (**C**) Comparison of wound closure rate between the combination of HA and ASC-EXOs (ASC-EXO + HA) treatment and HA alone. (**D**) Representative images of wound sites with each treatment on days 7, 14, 17, and 19. (**E**) Histological examination of skin tissue sections stained with hematoxylin and eosin (H&E) showing re-epithelialization (Re), inflammation (In), and granulation tissue (Gn). Enlarged images of each section are shown in the right panel, corresponding to the boxed area in the left panel. (**F**) Quantification of epithelialization score (0–3) indicating tissue repair. Scale bars represent 1 mm. The data are shown as the mean ± SD (*n* = 4) with significance at ** *p* < 0.01.

Standard wound excisions measuring 30 mm in length and width were generated as cross-sections in eight different sites in porcine skin for observation and analysis (Figure 2A). Topical treatment of porcine skin wounds was performed using a combination of HA and ASC-EXOs, or conjugated HA alone. Topical administration was conducted three times per week at various timepoints, and skin tissue collection was performed at the study endpoint of 3 weeks (Figure 2B). As shown in Figure 2C,D, topical treatment with the combination of HA and ASC-EXOs resulted in a higher wound closure rate compared to treatment with conjugated HA alone in porcine skin wounds. Although there was high variability in the closure rate, the ASC-EXO + HA group showed an approximately 10% increase in wound closure rate compared to the HA-alone group (Figure 2C,D). Histological examination of tissue sections stained with hematoxylin and eosin (H&E) revealed a statistically significant increase in re-epithelialization in wounds treated with the combination of HA and ASC-EXOs compared to conjugated HA alone-treated sites (Figure 2E,F).

**Figure 3:**
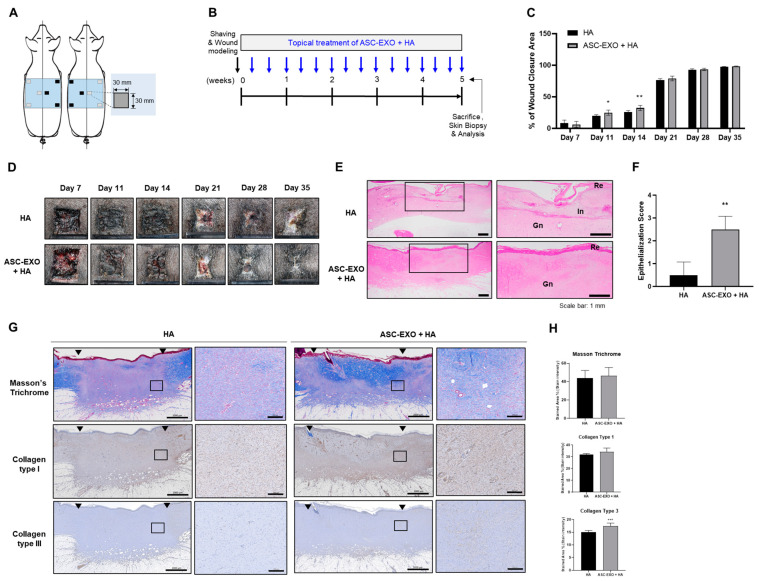
Effects of ASC-EXOs on wound closure, re-epithelialization, and collagen synthesis in an extended porcine acute wound healing model. (**A**) Schematic diagram illustrating the skin wounds and the topical treatments in two SPF mini pigs. (**B**) Schedule of wound modeling, topical treatment, and tissue collection. (**C**) Comparison of wound closure rate between the combination (ASC-EXO + HA) treatment and HA alone. (**D**) Representative images of wound sites with each treatment on days 7, 11, 14, 21, 28, and 35. (**E**) Histological examination of skin tissue sections stained with H&E showing re-epithelialization (Re), inflammation (In), and granulation (Gn). Enlarged images of each section are shown in the right panel, corresponding to the boxed area in the left panel. Scale bars represent 1 mm. (**F**) Quantification of epithelialization score (0–3) indicating tissue repair. (**G**) Tissue sections were stained with Masson’s trichrome for visualization of collagen fibers and immunohistochemistry (IHC) for collagen type I and type III. Arrows indicate the edges of the remaining scar. Enlarged images of each section are shown in the right panel (scale bars: 200 μm), corresponding to the boxed area in the left panel (scale bars: 2000 μm). (**H**) Quantification of stained tissue area indicating collagen fiber, collagen type I, and type III. Data in the graphs are presented as the mean ± SD (*n* = 6) with significance at * *p* < 0.05, ** *p* < 0.01, and *** *p* < 0.001.

To further support these findings, an extended experiment was performed with an observation period of 5 weeks. In this case, standard wound excisions were generated in six different sites in two porcine models to reduce selection bias and allow for better comparison (Figure 3A). Similarly, topical administration was conducted three times per week at various timepoints, wound closure was assessed every week, and skin tissue collection was performed at the study endpoint of 5 weeks for histological examinations (Figure 3B). As shown in Figure 3C,D, the combination of HA and ASC-EXO treatment showed significantly increased wound closure compared to the HA-alone group as measured on days 11 and 14. This difference was also evidenced by a statistically significant increase in re-epithelialization in wounds treated with the combination of HA and ASC-EXOs compared to HA alone (Figure 3E,F). Furthermore, the combination treatment exhibited a reduction in inflammation compared to the HA-alone group (Figure 3E). Although the combination of HA and ASC-EXOs did not increase type I collagen and collagen fiber, collagen type III, intriguingly, was higher in wound sites treated with the combination of HA and ASC-EXOs compared to the HA-alone group (Figure 3G,H), suggesting improved collagen deposition and tissue remodeling in the combination-treated group. Together, these results show that the combination of HA and ASC-EXOs significantly increased wound closure, re-epithelialization, and collagen III compared to HA alone in a porcine wound healing model, indicating a potential beneficial effect of the ASC-EXOs on wound healing by improving collagen deposition and tissue remodeling.

### 2.3. Effects of the Combination of HA and ASC-EXOs in a Mouse Dermal Filler Model

**Figure 4:**
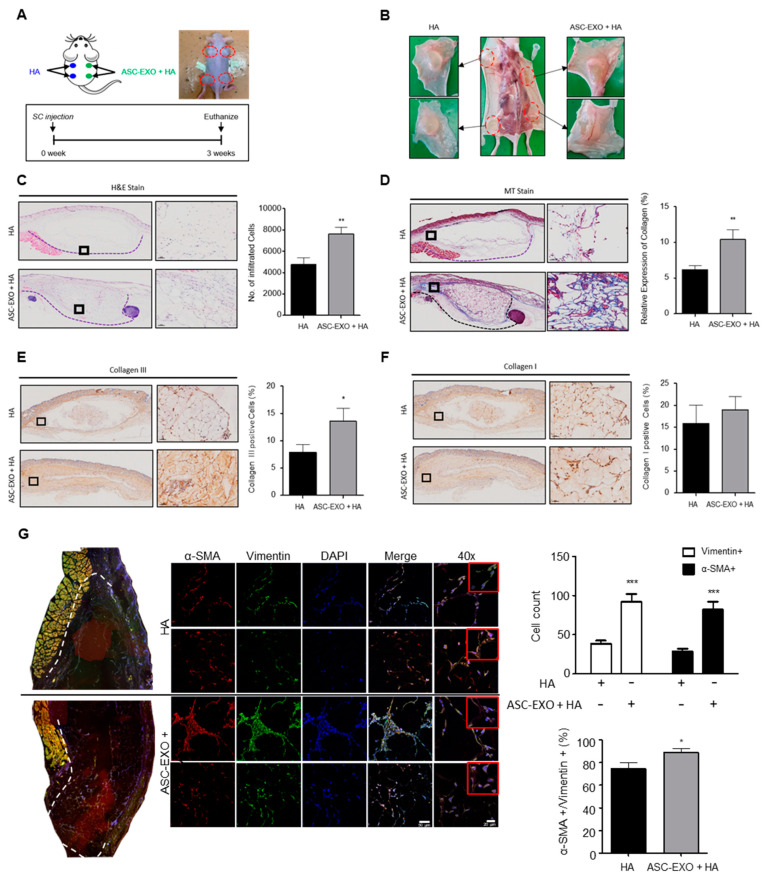
Effects of ASC-EXOs on tissue regeneration in a mouse dermal filler model. (**A**) Schematic illustration showing the subcutaneous injection of HA or ASC-EXO + HA into the back of SKH1-hairless mice (6 weeks old, *n* = 4). (**B**) Representative images of explanted tissue graft 3 weeks after injection. (**C**) Histological evaluation of the grafts stained with H&E and quantification of infiltrated cells at 3 weeks post injection. Data are presented as the mean ± standard deviation (SD) with significance at ** *p* < 0.01. (**D**) Masson’s trichrome staining to visualize collagen fiber in blue and IHC staining for collagen type I (**E**) and type III (**F**). Data collected from three different areas of every slide are presented as the mean ± standard error of the means (SEM) with significance at * *p* < 0.05 and ** *p* < 0.01. Scale bars for (**C**,**D**), 50 µm. (**G**) Histological evaluation of the grafts for expression of α-SMA and vimentin. Representative IF images are shown, and the number of α-SMA-positive or/and vimentin-positive cells was quantified in three different areas of every tissue slide. Data are shown as the means ± SEM with significance at * *p* < 0.05 and *** *p* < 0.001. Scale bars represent 50 µm and 20 µm for the 20× and 40× magnification images, respectively.

To assess the biological effects of ASC-EXOs on murine skin in a euthymic and immunocompetent environment, a mouse dermal filler model was employed. The combination of HA and ASC-EXOs or HA alone was subcutaneously injected into four dorsal sites of SKH1-hairless mice, and, after 3 weeks, the tissues surrounding the transplanted grafts were collected for histological analysis (Figure 4A,B). Notably, visible differences were observed in the tissues treated with the combination of HA and ASC-EXO-treated tissues, which showed thicker tissue layers and increased vascularization compared to the sites injected with HA alone (Figure 4B). H&E staining revealed increased cell infiltration in the combination-treated group, nearly twice as much as in the HA-alone group (Figure 4C). Masson’s trichrome staining further demonstrated a higher amount of collagen fiber in the tissues transplanted with the combination of HA and ASC-EXOs compared to the HA-alone sites (Figure 4D). IHC staining for collagen I and III showed consistent results with the porcine wound healing model, indicating higher expression of type III collagen in the combination-treated group with no statistical significance observed for collagen type I (Figure 4E,F). Additionally, immunofluorescence (IF) staining for α-SMA and vimentin in the transplanted tissues was performed to examine fibroblast differentiation. As shown in Figure 4G, the combination of HA and ASC-EXOs increased the number of vimentin-positive cells, as well as α-SMA-positive cells, compared to HA alone, indicating approximately a two-fold increase in myofibroblast infiltration. Furthermore, merging the staining for vimentin and α-SMA indicated that most vimentin-positive cells expressed α-SMA, and the percentage ratio of α-SMA-positive to vimentin-positive cells was significantly higher in the combination of HA and ASC-EXO-treated transplants compared to HA alone, suggesting enhanced differentiation into myofibroblasts (Figure 4G). Overall, these findings suggest that ASC-EXOs may promote tissue regeneration by increasing the infiltration and differentiation of myofibroblasts and inducing ECM remodeling in the mouse dermal filler model. 

## 3. Discussion

The present study investigated the effects of ASC-EXOs on wound healing and tissue regeneration in both in vitro and in vivo settings. ASC-EXOs, when used alone or in combination with HA, demonstrated positive effects on cell proliferation, migration, and ECM remodeling in HDFs in vitro, as well as enhanced wound closure and tissue regeneration in porcine and mouse models in vivo. ASC-EXO treatment increased cell proliferation, up-regulated gene expression related to wound healing, and increased collagen production in HDFs, suggesting the potential for ECM remodeling.

HA, a major component of the skin ECM, is widely recognized for its valuable properties involved in inflammation regulation, angiogenesis promotion, and tissue regeneration. It is commonly used as a biomaterial for wound dressings due to its inherent characteristics of biocompatibility, biodegradability and hydrophilicity [18]. In the porcine wound healing model (Figure 2 and Figure 3), the combination of HA and ASC-EXOs demonstrated several favorable outcomes compared to HA alone. Firstly, the combination treatment resulted in higher rates of wound closure, indicating improved healing efficiency. Additionally, the combination treatment led to a reduction in inflammation and increased re-epithelialization, further supporting its beneficial effects. Furthermore, the combination of HA and ASC-EXOs showed an increase in type III collagen while no significant changes were observed in type I collagen and collagen fiber. This alteration suggests a decrease in the ratio of type I to type III collagen, which is associated with scarless wound healing. Although the specific mechanisms and molecular signals involved in scarless fetal wound healing are not fully understood, it is widely recognized that increased HA levels, reduced inflammation, and alterations in the type I/III collagen ratio are distinguishing features of scarless wound healing compared to scarring in adult wounds [23]. Taken together, these findings indicate that the combination of HA and ASC-EXOs holds great potential for promoting less-scar wound healing compared to the HA alone in this porcine wound healing model. 

Moreover, HA is commonly used as a dermal filler component in cosmetic procedures to address wrinkles and enhance skin volume [17]. Recent studies have provided insights into the mechanisms underlying the long-lasting filling effect of HA, demonstrating its ability to activate fibroblasts and promote efficient collagen production and ECM formation [17]. In the mouse dermal filler model, the combination of HA and ASC-EXOs resulted in thicker tissue layers, increased vascularization, elevated levels of type III collagen, higher collagen fiber deposition, and enhanced infiltration of myofibroblasts (Figure 4). These findings collectively support the notion that the addition of ASC-EXOs contributes to enhanced tissue regeneration by activating fibroblasts and promoting angiogenesis.

Importantly, ASC-EXOs did not show any signs of toxicity or adverse effects in both the in vivo experiments conducted in the porcine and mouse models. These observations suggest that incorporating ASC-EXOs into the HA-based treatment regimen holds promise for augmenting wound healing and tissue regeneration processes, providing additional benefits beyond the effects of HA alone.

Exosomes play crucial roles in intercellular communication and have demonstrated therapeutic effects in various areas, including skin and bone regeneration and cardiac function recovery. However, their clinical application has been limited due to challenges such as low targeting ability and a short half-life in circulation [24,25]. To overcome these limitations, researchers have developed biodegradable or highly porous hydrogels as delivery systems for exosomes, enabling sustained therapeutic effects [24]. HA-based hydrogels, as an important component of the ECM and a commonly used biomaterial in wound dressings and dermal fillers, have also been utilized as an exosome delivery system [22,24,25]. These hydrogels protect exosomes from degradation, prolong their bioactivity, and have shown positive effects on wound healing and bone regeneration [22,24,25]. The improved effects observed in our in vivo experiments using the combination of HA and ASC-EXOs compared to in vitro experiments using ASC-EXOs alone can be attributed to these factors. The HA-based hydrogel serves as an effective carrier for ASC-EXOs, enhancing their stability and promoting their therapeutic potential. These findings suggest that the combination of HA and ASC-EXOs holds promise for both clinical and regenerative aesthetic applications in skin repair and regeneration. By addressing the challenges associated with exosome delivery, this approach may provide a valuable option for improving outcomes in these areas. 

Wound healing in the skin is a complex and highly regulated process involving distinct phases: hemostasis, inflammation, proliferation, and remodeling. These phases collaborate to restore the integrity and functionality of the skin [11]. Epithelialization, a key process during the proliferation phase, is crucial for wound closure. While epidermal stem cells and keratinocytes play essential roles in this process by proliferating and migrating to cover the wound bed, re-epithelialization is not solely governed by epidermal cells [11,26]. Multiple proinflammatory cytokines and growth factors secreted by immune cells and fibroblasts contribute to the regulation of keratinocytes and epidermal stem cells, necessitating intricate interactions and crosstalk between these cell types to orchestrate an effective and timely wound healing response [11,26]. Fibroblasts are critical in wound healing as they secrete collagen, which forms the foundation of granulation tissue. Granulation tissue facilitates angiogenesis, the formation of new blood vessels crucial for supplying fluid, oxygen, nutrients, and immune cells to the healing wound [11,26]. The interconnected network of keratinocytes, fibroblasts, inflammatory cells, and epidermal stem cells is vital for achieving successful wound closure [11,26]. In our in vivo wound healing model, the combination of HA and ASC-EXOs demonstrated several favorable outcomes: higher rates of re-epithelialization and wound closure, increased levels of type III collagen and granulation tissue, and a reduction in inflammation (Figure 3). Additionally, ASC-EXOs promoted cell proliferation, migration, and the expression of genes involved in ECM remodeling in HDFs (Figure 1). Notably, ASC-EXOs have been reported to activate epidermal stem cells, induce keratinocyte differentiation, and enhance keratinocyte proliferation, survival, migration, and collagen secretion [11]. These findings collectively highlight the multifaceted effects of ASC-EXOs on various cell types crucial for wound healing processes, inflammation, granulation, and epithelialization. A comprehensive understanding of the complex interplay between these cellular components and their regulatory factors is paramount in unraveling the molecular mechanisms through which ASC-EXOs enhance wound healing and tissue regeneration. Furthermore, investigating the protein components and RNA cargo of ASC-EXO is imperative for comprehending exosome-mediated wound healing. MSC-derived exosomes possess regenerative and anti-inflammatory properties, likely due to their unique cargo that differs from other cell types. Modulating exosome cargo, such as altering the expression of miR-29a in MSC-derived exosomes, has demonstrated the ability to inhibit fibrosis and enhance fibroblast migration and proliferation [27]. Understanding the inheritable and noninheritable components of exosomes may aid in predicting their therapeutic effects in tissue regeneration. Elucidating the intricate mechanisms underlying the effects of ASC-EXOs will identify potential intervention targets and promote more efficient and successful wound healing outcomes.

Exosomes offer a simpler structure and nontoxic properties compared to stem cells, which have complex structures and associated safety concerns, presenting an opportunity to develop novel cell-free treatments for various health conditions. Clinical trials are currently underway to evaluate the utility of exosomes for diagnostic and therapeutic purposes in diseases such as cancer, type I diabetes, and inflammatory bowel disease, which may provide valuable insights into human responses and regeneration processes [28]. Importantly, repeated intravenous dosing of MSC-derived exosomes in clinical settings has not been associated with any reported toxicity [29]. Similarly, the present study did not observe any adverse effects of ASC-EXOs in in vivo experiments conducted in two different species, supporting the safety of MSC-derived exosomes. However, detailed follow-up in clinical trials will be important to further validate their safety and efficacy. 

Taken together, these results provide important insights into the potential therapeutic and cosmetic applications of ASC-EXOs in wound healing and tissue regeneration. Further studies are warranted to elucidate the underlying mechanisms of ASC-EXO-mediated effects on wound healing and tissue regeneration, as well as to evaluate its safety and efficacy in clinical settings. Overall, the findings from this study suggest that ASC-EXOs, alone or in combination with HA, have the potential to be used as a novel therapeutic approach for wound healing and tissue regeneration in various clinical applications.

## 4. Materials and Methods

### 4.1. Culture and Characterization of ASCs

Human adipose tissue from a healthy donor was collected by ExoCoBio Inc. (Seoul, Republic of Korea) with the approval of the Institutional Review Board of CHA University Medical Center, Republic of Korea, and assessed according to the guidelines of the Korean Ministry of Food and Drug Safety (MFDS). ASCs were isolated from the adipose tissue and sub-cultured with MEM-α (Gibco, Grand Island, NY, USA) containing 10% fetal bovine serum (FBS) (Gibco, Grand Island, NY, USA) at a density of 3000 cells/cm^2^, in a humidified atmosphere containing 5% CO_2_ at 37 °C. Cells were disassociated using 0.25% Trypsin-EDTA (Gibco, Grand Island, NY, USA), and then washed with Dulbecco’s phosphate-buffered saline (DPBS) (Gibco, Grand Island, NY, USA); cell stocks of passage 4 were stored in liquid nitrogen. The quality of ASCs was evaluated through tests for sterility, mycoplasma, cell viability, endotoxins, and viruses, and ASCs were characterized for surface-marker expression and trilineage differentiation potential according to the criteria described by the International Society of Cellular Therapy [30].

### 4.2. ASC-EXO Isolation and Characterization

To obtain ASC-conditioned medium (CM), a vial of ASC stock was thawed and sub-cultured with gradually increasing culture scales in a T175 flask and a one- or two-layered Cell Factory (Thermo Fisher Scientific, Carlsbad, CA, USA) until passage 7 at 37 °C and 5% CO_2_. At passage 7, ASCs were plated at a density of 6000 cells/cm^2^ in 10-layered Cell Factories and cultured up to 90% confluency in MEM-α containing 10% FBS at 37 °C and 5% CO_2_. The ASCs were washed three times with DPBS to remove FBS and supplemented with serum- and phenol-red-free MEM-α. The cells were further incubated for 24 h at 37 °C and 5% CO_2_ before ASC-CM was collected. ASC-EXOs were isolated from ASC-CM using the tangential flow filtration (TFF)-based ExoSCRT™ technology as previously described [31]. Briefly, ASC-CM was filtered through a 0.22 μm polyethersulfone membrane filter (Merck Millipore, Billerica, MA, USA), and then concentrated by TFF with a 500 kDa molecular weight cutoff filter. The concentrated ASC-CM was further diafiltrated with appropriate volumes of PBS, and then stored at −80 °C as small aliquots in sterile polypropylene tubes for further use. Characterization of the ASC-EXOs was performed according to the Minimal Information for Studies of Extracellular Vesicles 2018 (MISEV2018) recommended by the International Society for Extracellular Vesicles [32]. 

### 4.3. Human Dermal Fibroblasts, Cell Proliferation, and Migration

HDFs were purchased from the American Type Culture Collection (ATCC; Manassas, VA, USA). HDFs were cultured in Dulbecco’s modified Eagle medium (DMEM; Gibco, Grand Island, NY, USA) supplemented with 10% FBS and 1% penicillin/streptomycin, and incubated at 37 °C with 5% CO_2_. HDFs proliferation was evaluated using a CCK-8 assay kit following the manufacturer’s instructions (Dojindo, Kumamoto, Japan). HDFs were seeded into 48-well plates at a density of 20,000 cells/well and incubated for 24 h. The cells were then treated with ASC-EXOs in serum-deficient medium (1% FBS) or positive control (complete growth medium with 10% FBS) for 24 h, and cell proliferation was measured using CCK-8 assays. Absorbance was measured at 450 nm using a microplate reader. HDF migration was measured using scratch wound healing assays with the Incucyte Live-Cell Analysis System (Sartorius, Goettingen, Germany). HDFs were cultured to 100% confluency on a 96-well plate, and the assay was performed according to the manufacturer’s manual. Briefly, a straight scratch was created in the middle of the well to remove cells. Wells were then washed once with DPBS, and fresh medium with or without ASC-EXOs in serum-free medium (no serum) or positive control (complete growth medium) was added to the wells. The well plate was marked and placed in the Incucyte to ensure that images were taken in the same position, and cell migration progress was monitored every hour.

### 4.4. Evaluation of Collagen Production—PIP Assays

The production of procollagen type 1 C-peptide (PIP) was measured using a PIP enzyme immunoassay kit following the manufacturer’s instructions (Takara, Shiga, Japan). HDFs were seeded in a 24-well plate and treated with ASC-EXOs in serum-free medium or positive control (complete growth medium) for 24 h. The PIP contents were measured using a microplate spectrophotometer at 450 nm. 

### 4.5. Total RNA Extraction and Real-Time Quantitative RT-PCR

Total RNA was extracted from HDFs treated with ASC-EXOs in serum-free medium or positive control (complete growth medium) using an RNeasy kit (Qiagen, Venlo, Netherlands) following the manufacturer’s instructions. The purified RNA was then resuspended in Rnase-free water and quantified using spectrophotometry. Aliquots of total RNA were stored at −80 °C immediately after extraction. For reverse transcription, 1 µg of extracted RNA was used with PrimeScript Reverse Transcriptase (Takara, Shiga, Japan), followed by real-time quantitative reverse transcription polymerase chain reaction (qRT-PCR) to quantify mRNA expression of genes encoding collagen type I and III, α-SMA, hFGF2, and elastin. Glyceraldehyde 3-phosphate dehydrogenase (*GAPDH*) was used as an internal control to normalize gene expression levels. The real-time qRT-PCR reaction was performed using 1 µL of total cDNA, 0.5 µL of each primer, and an RT-PCR kit with SYBR Green (KaPa Biosystems, Wilmington, MA, USA) according to the manufacturer’s protocols (45 cycles consisting of 3 min at 95 °C, 3 s at 95 °C, and 30 s at 60 °C). The efficiency and specificity of each primer set were confirmed with a standard curve and melting profile analysis. Specific primers were used as shown in Table 1.

### 4.6. HA/Hydrogel Preparation and ASC-EXO Encapsulation

The HA filler used in this study was Celosome Aqua product (HA concentration of 2.4%) made by ExoCoBio Inc. ASCs-EXOs (100 µg) were encapsulated in HA solution, and the mixture was blended and stored at 4 °C.

### 4.7. In Vivo Porcine Wound Healing Experiments

All procedures were approved by the Institutional Animal Care and Use Committee of Knotus (Incheon, Republic of Korea) (IACUC 19-KE-222). SPF micro pigs (male, weighing 20 kg) were purchased from CRONEX.CO., LTD (Seoul, Republic of Korea). A full-thickness wound measuring 30 mm × 30 mm was created on the pig model as previously described [33]. The pigs were randomized into two different groups: (1) HA group, treated with 250 µL of HA; (2) HA + ASC-EXO group, treated with ASC-EXOs (4.0 × 10^10^ particles/mL) dissolved in 250 µL of HA. The treatments were topically applied to the wound sites a total of nine times over 3 weeks or 15 times over 5 weeks (three times a week). Postoperative pigs were housed individually. The wound areas were measured on days 0, 7, 14, 17, and 19 or days 7, 11, 14, 21, 28, and 35 in the extended experiments. Skin tissue including the wound area was collected at the study endpoint of 3 or 5 weeks for histological analysis. The wound closure rate was calculated using the following formula: wound healing rate = (W_0_ − W_d_)/W_0_ × 100%, where W_0_ is the wound area on day 0, and W_d_ is the wound area on day d post treatment.

### 4.8. In Vivo Mouse Dermal Filler Experiments

All procedures were approved by the Institutional Animal Care and Use Committee of Kyung Hee University Medical Center (Seoul, Republic of Korea) (IACUC 2019-018). SPF SKH1-hairless mice (6 weeks old, weighing 28–35 g) were purchased from ORIENT BIO Inc. (Seongnam, Republic of Korea). Mice were divided into two groups: (1) HA group, treated with 200 µL of HA; (2) HA + ASC-EXO group, treated with ASC-EXOs (1.4 × 10^9^ particles/mL) dissolved in 200 µL of HA. Each treatment was subcutaneously injected into two dorsal sites of SKH1-hairless mice, and, after 3 weeks, the transplanted grafts were collected for histological analysis.

### 4.9. Histological Analysis, Immunohistochemistry, and Immunofluorescence Staining

Tissue sections of 5 µm thickness were mounted on slides for histological analysis. H&E and Masson’s trichrome staining was used to visualize the pathological changes in the rejuvenated tissue and collagen formation at various healing times. For IHC, the dewaxed sections were washed with PBS, and endogenous peroxidase activity was quenched by immersion in 2% (*v*/*v*) hydrogen peroxide for 5 min. Antigen retrieval was performed by incubation with sodium citrate buffer for 30 min. After rinsing with PBS, the sections were blocked with 1.5% goat serum at room temperature for 30 min. Then, the sections were incubated with primary antibodies against collagen I (1:200; ab6308, Abcam, Cambridge, MA, USA) and collagen III (1:200; ab23445, Abcam, Cambridge, MA, USA). IHC staining was developed using the DAB substrate system (DAKO, Glostrup, Denmark). Tissues stained with H&E, Masson’s trichrome, and IHC were observed, and images were acquired using an optical microscope (BX53; Olympus, Tokyo, Japan). The mean density of positively stained regions was quantified using ImageJ 1.53k software (NIH, Bethesda, MD, USA). For IF staining, primary antibodies against vimentin (1:50; V4630, Sigma, St. Louis, MO, USA) or α-SMA (1:200; ab5694, Abcam, Cambridge, MA, USA) were used, followed by incubation with isotype-specific secondary antibodies conjugated to Alexa Fluor 488 or Alexa Fluor 546 (1:1000; Thermo Scientific, Carlsbad, CA, USA). Sections were then incubated with 4,6-diamidino-2-phenylindole (DAPI) for 1 min to visualize nuclei. Images of IF staining were captured using a confocal microscope (LSM700, Carl Zeiss, Jena, Germany).

### 4.10. Statistical Analysis

Statistical analysis was conducted using GraphPad Prism 5 software. One-way ANOVA was used when comparing more than two groups of samples with one condition, while two-way ANOVA was used for comparisons involving more than two conditions. Post hoc Bonferroni’s correction for multiple comparisons was applied after ANOVA analysis. A significance level of *p* < 0.05 was considered statistically significant, denoted as follows: * *p* < 0.05, ** *p* < 0.01, *** *p* < 0.001, and **** *p* < 0.0001. Data from at least three independent experiments were presented as the mean ± SD.

## Figures and Tables

**Table 1:** Primer sequences for real-time quantitative RT-PCR analysis of target genes.

	Forward Primer (5′–3′)	Reverse Primer (5′–3′)
*COL1A1*	AGTGGTTTGGATGGTGCCAA	GCACCATCATTTCCACGAGC
*COL3A1*	TCGAGGCAGTGATGGTCAAC	TCCTGGGATGCCATTTGGTC
*ELN*	GGGTTGTGTCACCAGAAGCA	CAACCCCGTAAGTAGGAATGC
*bFGF*	AGAAGAGCGACCCTCACATCA	CGGTTAGCACACACTCCTTTG
*α-SMA*	CTATGAGGGCTATGCCTTGCC	GCTCAGCAGTAGTAACGAAGGA
*GAPDH*	CTTTGGTATCGTGGAAGGACTC	GTAGAGGCAGGGATGATGTTCT

## Data Availability

Not applicable.
